# Genome-Wide Analysis of Innate Susceptibility Mechanisms of *Escherichia coli* to Colistin

**DOI:** 10.3390/antibiotics11111668

**Published:** 2022-11-21

**Authors:** Muhammad Yasir, A. Keith Turner, Sarah Bastkowski, Martin Lott, Emma R. Holden, Andrea Telatin, Andrew J. Page, Mark A. Webber, Ian G. Charles

**Affiliations:** 1 Quadram Institute Bioscience, Rosalind Franklin Road, Norwich NR4 7UQ, UK; 2 Norwich Medical School, Norwich Research Park, Colney Lane, Norwich NR4 7TJ, UK

**Keywords:** TraDIS-Xpress, colistin, resistance mechanisms, *Escherichia coli*

## Abstract

Colistin is an antibiotic that has seen increasing clinical use for the treatment of human infections caused by Gram-negative pathogens, particularly due to the emergence of multidrug-resistant pathogens. Colistin resistance is also a growing problem and typically results from alterations to lipopolysaccharides mediated by phosphoethanolamine (pETn) transferase enzymes which can be encoded on the chromosome, or plasmids. In this study, we used ‘TraDIS-Xpress’ (**Tra**nsposon **D**irected **I**nsertion site **S**equencing with e**xpress**ion), where a high-density transposon mutant library including outward facing promoters in *Escherichia coli* BW25113 identified genes involved in colistin susceptibility. We examined the genome-wide response of *E. coli* following exposure to a range of concentrations of colistin. Our TraDIS-Xpress screen confirmed the importance of overexpression of the two-component system *basSR* (which regulates pETn transferases) but also identified a wider range of genes important for survival in the presence of colistin, including genes encoding membrane associated proteins, DNA repair machinery, various transporters, RNA helicases, general stress response genes, fimbriae and phosphonate metabolism. Validation experiments supported a role in colistin susceptibility for novel candidate genes tested. TraDIS-Xpress is a powerful tool that expands our understanding of the wider landscape of genes involved in response to colistin susceptibility mechanisms.

## 1. Introduction

Antibiotic-resistance is a global challenge to society, and treatment of multidrug-resistant (MDR) bacteria relies on a small number of critical antibiotics [1]. Colistin is a cyclic peptide with lipophilic and hydrophilic moieties that interact with the outer membrane of Gram-negative bacteria; currently it remains active against most antibiotic-resistant Gram-negative human pathogens [2].

Colistin has been used widely in veterinary medicine and, in some parts of the world, as a growth promoter in animal feed [3]. Initially it was not widely used in human medicine due to concerns over nephrotoxicity and neurotoxicity [4]. However, due to the emergence of bacterial pathogens resistant to multiple antibiotics, colistin has been used as a drug of last resort in human medicine [5].

Colistin resistant bacterial pathogens have emerged in animals, including chickens, and are increasingly being found in humans [6]. The most common mechanism for colistin resistance is the modification of lipopolysaccharides (LPS) in the outer membrane via the addition of phosphoethanolamine (pEtN) by pEtN transferase enzymes from phosphatidylethonalamine (PE), which lowers the surface negative charge and reduces the affinity for colistin binding [7]. 

Before 2015, colistin resistance mechanisms were solely reported due to chromosomally encoded pEtN transferases; however, a mobile pEtN transferase gene (*mcr*-1) was then found on a conjugative plasmid in *E. coli* [8]. Since then, *mcr*-1 has been reported in many different Gram-negative bacterial species and from various locations globally [6,9]. 

Modification of LPS is often achieved by mutations in the two-component regulatory systems (e.g., *basSR*, *phoPQ* and *parRS*) responsible for the regulation of a whole battery of genes, including pEtN transferases. For example, activation of a PhoPQ two-component system results in increased *mgrB* transcription, which then negatively suppresses the phosphorylation of PhoP. Mutations in *mgrB* prevent this negative feedback suppression of phosphorylation of PhoP, and, as a result, can alter colistin susceptibility [10]. Furthermore, efflux pump overexpression, porin loss and upregulation of capsular biosynthesis all have a role in colistin susceptibility [11,12]. 

These known resistance mechanisms have been identified over a prolonged period by analysis of resistant isolates or defined mutants with target genes inactivated in the laboratory. There have also been studies using transcriptomics to understand colistin susceptibility mechanisms; these studies have provided important insights into a wider group of genes responsive to colistin-induced stress [13]. High-throughput transposon mutagenesis coupled with sequencing is another approach that is proving useful for elucidating the roles of genes involved in the overall cellular response to a particular stress. Recent studies using this approach have reported a central role for the *pmrAB* two-component system in colistin resistance in *Klebsiella pneumoniae* and *Acinetobacter baumannii* [14]. While *E. coli* is one of the most studied model organisms available, there have been no genome-wide transposon mutagenesis studies to determine the role(s) of all genes implicated in colistin susceptibility.

In this study, we used a high-density transposon mutant library of *E. coli* to determine the role played by genes following exposure to colistin stress. We used our recently described new method, ‘TraDIS-Xpress’ (**Tra**nsposon **D**irected **I**nsertion site **S**equencing with e**xpress**ion), in which the transposon includes an outward-transcribing inducible promoter [15]. This technology enables both inactivation of genes and changes in gene expression to be evaluated following an increase or decrease in mutants from any population pool. This allows us to assay roles for all genes in a condition, including essential genes which cannot be inactivated but where changes in expression can be identified as being important. We carried out a genome wide survey of all genes in *E. coli* that were important for survival and growth following exposure to four levels of colistin stress. 

## 2. Results

### 2.1. Effect of Colistin Stress on the E. coli Mutant Library

For *E. coli* BW25113, the minimum inhibitory concentration (MIC) of colistin was experimentally determined as 0.5 μg/mL. Using this MIC of colistin as a reference point, 30 TraDIS-Xpress experiments, including controls, were conducted (Table S1) and the insert sites of all transposons in each experiment were determined using Illumina high-throughput sequencing. The mutants recovered in each experiment vary a lot depending on the colistin stress level exposure (Table S2). Survival of mutants following exposure to a range of colistin concentrations was used to identify loci important for susceptibility mechanisms. Figure S1 shows the high degree of similarity and reproducibility between replicates, as shown by the correlation values (Table S3). A total of 50 genes of interest were consistently identified under multiple colistin exposure conditions mostly associated with lipid biosynthesis regulation, transcription and translation regulation, transporters, and membrane related genes (Table 1). A few genes were only significantly different in one replicate, or single stress condition. We considered those genes where good statistical support for a role in colistin susceptibility was consistently observed in multiple conditions and replicates as strong candidates for follow up.

### 2.2. Effect of Colistin on Cell Membranes

**Figure 1:**
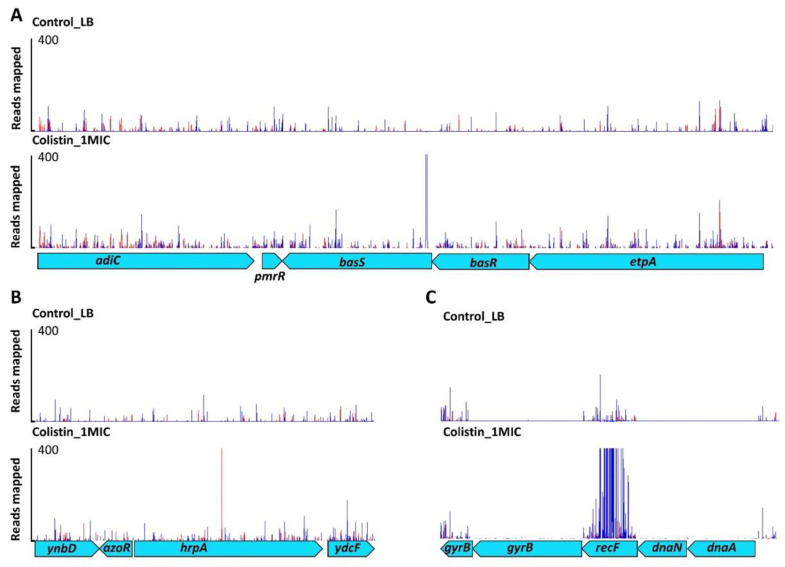
Differential selection and expression of *basS*, *hrpA* and *recF* transposon mutants. Identification of known targets was performed using the TraDIS-Xpress approach incorporating an inducible outward-facing promoter which identifies the impact of both essential and non-essential genes on survival and growth. A genetic map of the relative gene positions is shown at the bottom of the panel. Above this, each row of vertical red or blue lines (plotted with red behind) indicates the position of mapped reads; the height of the bar represents the relative number of reads mapped and the *Y*-axis scale indicates a scale of 400, representing up to 400 reads mapped in these plots being visible. Red indicates transposon insertions, wherein the transposon-encoded kanamycin resistance gene is oriented 5′ to 3′ left to right, and blue indicates the opposite (right to left) orientation. The top row plot shows untreated control culture and the bottom row is culture grown in the presence of colistin stress. (**Panel A**) shows inserts predicted to result in an up-regulation of *basS* in the presence of colistin. (**Panel B**) shows the up-regulation of the *hrpA* domain compared with the control. (**Panel C**) shows the knockout of *recF*.

Overnight growth in colistin selected for a large expansion of mutants with transposon insertions within the *basS* gene. Most of these insertions were located within the 5′ end of *basS*, and all these insertions were present within a few bp region, with the highest number located 36 bp from the start of the gene. In addition, all the insertions were oriented such that the outward-transcribing transposon promoter would transcribe *basS* from this site (Figure 1A). At 2× MIC exposure, the majority of insert sites present in the population were located in a 9 bp region (over half the total population of reads generated). This indicates that increased expression of *basS* confers a significant selective advantage at this concentration of colistin in vitro. The outward facing promoter in transposon modulates the expression of essential genes by overexpression as *basS* (Figure S2). Our data show that colistin strongly selects Tn mutants that have insertions in the operon that includes *basSR* and *eptA*. The *basS* gene appears to be essential at high concentrations of colistin but there were insertions after 36 bp from the start of the gene (Figure 1A). TraDIS-Xpress has the potential to identify important regions within the genes (at base-pair resolution) and this is clearly demonstrated as all the over-expression insertions are 36 bp inside the gene from the 5′ end of the gene, suggesting that the first 12 amino acids of BasS may be dispensable for colistin resistance. 

The lipid biosynthesis pathway (*waaB*, *waaC*, *waaG*, *waaP*, *waaR*, *waaS*, *waaU*) appeared to be protected at the 1× MIC and 0.5× MIC concentrations of colistin, and the whole operon was identified as important for survival under different levels of colistin stress, indicating its importance in survival of the mutants.

Outer membrane associated proteins were generally identified as important contributors to survival under colistin stress, over-production of fimbriae helped survival under high colistin stress, and protection of the outer membrane encoding genes *bhsA* and *ompA* was also observed to be important under colistin stress. 

### 2.3. DNA Housekeeping Genes Associated with Colistin Susceptibility

Many mutants were identified with transposon insertions within an ATP-dependent RNA helicase gene, *hrpA*. All these insertions were located centrally in the 3.9 kb open reading frame (ORF), within a 15 bp region (14 different insertion sites were seen) (Figure 1B). The outward-facing transposon promoter was capable of transcribing the 3′ end of the gene after this insertion site, which raises the possibility that part of the protein after the insertion site may remain functional (Figure 1B). At 2× MIC for colistin, around 1 in 10 of all recovered mutants had transposon insertions located in this 15 bp region. Transposon insertions outside of this small region within *hrpA* did not confer a selective advantage at any of the colistin concentrations tested. 

**Figure 2:**
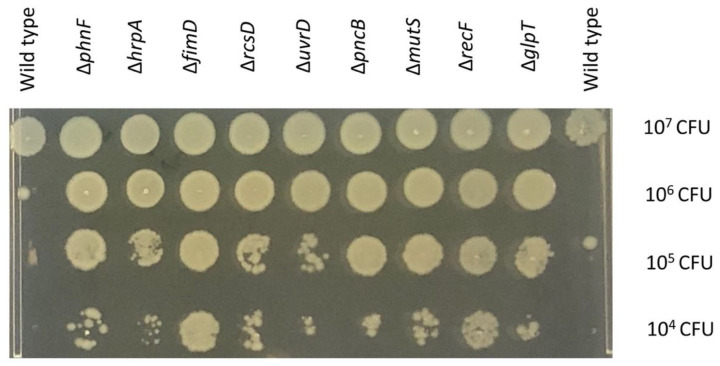
Validation of the contributions of defined mutants to colistin sensitivity. Mutants and the wild type were serially diluted (10^7^, 10^6^, 10^5^, 10^4^ colony-forming units from top row down) onto an MH agar plate containing 0.5 μg/mL of colistin and incubated overnight at 37 °C.

Several other novel loci were identified in addition to *hrpA*. At various concentrations of colistin, other genes relating to DNA repair protein machinery (*recF* and *uvrD*) contributed to colistin susceptibility (Figure 1C). These genes are part of the bacterial DNA repair machinery but, unusually, their inactivation was predicted to aid survival under colistin stress [16]. These results were validated using defined mutants from the Keio collection, where deletions in the *recF* and *uvrD* genes were less sensitive to colistin than the wild type (Figure 2). 

### 2.4. Small RNA Dependent Regulation

Several mutants with insertion sites within sRNA genes (*cmoB, hfq, ohsC, oxyS, rne, rydC*) appeared to be important for susceptibility under colistin stress. Tn insertions in these genes had variable roles; some were protective, and some were detrimental in the presence of colistin, suggesting that downstream impacts from these regulators is important. 

### 2.5. GlpT Transporter and Phosphonates

Two transporters (*glpT*, *ygcS*) were implicated as having roles in colistin sensitivity, and inactivation of both was beneficial under colistin stress (Table 1). Two other loci that appeared to be significant for colistin susceptibility in our experiment were the *phn* and *fim* operons involved in phosphonate metabolism and adhesion, respectively. Our experiment predicted that knock out of these genes would be beneficial for cell survival during colistin stress, and this was again confirmed by analysis of defined Keio mutants. These operons may not be involved directly in the interaction with colistin but might affect the charge status of the outer membrane, which is important for colistin attachment. 

### 2.6. Validation of the Potential Genes

As our TraDIS-Xpress data report that an overexpression of *basS* gene provides an advantage in surviving under colistin stress (Table 1), we hypothesize that more copies or overexpression of *basS* gene should provide better survival chances in colistin stress. We transformed the *E. coli* BW25113 strain with pJMA/*basS* or pJMA vector and observed the overnight growth under colistin stress and different levels of induction with rhamnose for overexpression of *basS*. *E. coli* BW25113 containing pJMA/*basS* survived better as compared to *E. coli* BW25113 containing an empty vector as indicated by TraDIS-Xpress data (Figure S3). We also selected a battery of Keio mutants important for colistin survival and compared the growth of Keio mutants to the wild type. The mutants survived better in the presence of colistin as compared to the wild type (Figure 2). We also tested the susceptibility of some other selected mutants to different colistin concentrations. This showed that a *basR* mutant had a clear phenotype, as expected from TraDIS-Xpress data where there was a very strong signal for this gene. Other mutants which had weaker signals in the data, including *ompA*, *dnaG* and *surA*, did not exhibit a clear phenotype on agar dilution susceptibility testing (Figure S4). This is likely to be due to them having a relatively smaller impact on fitness in the presence of colistin, which does not translate into a large susceptibility difference when tested in isolation.

## 3. Discussion

**Figure 3:**
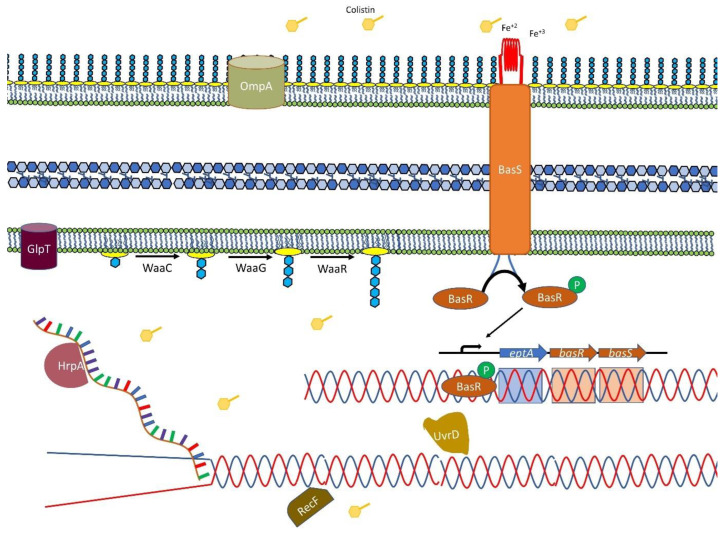
Pathways implicated in LPS modification and colistin resistance. This illustration shows how the two-component system BasSR is activated by iron or colistin, which then activates the *etpA* and *basSR* operon. Overexpression of *basSR* leads to modification of the LPS moiety via addition of the positively charged molecules L-Ara4-N and PEtN. The LPS biosynthesis pathway (waa) genes are protected under colistin stress, indicating an essential role. The illustration depicts UvrD and RecF and RNA helicase HrpA representing DNA and RNA associated proteins important in colistin stress.

Using TraDIS-Xpress, we observed that changes to the BasSR system (which controls pEtN transferase expression) were most prominently selected after exposure to colistin. The BasSR two-component system is known to mediate colistin resistance and constitutes a transmembrane protein that acts as a sensor for differences in concentrations of divalent ions (particularly iron) and changes in pH [17]. BasS phosphorylates the BasR component of the system in response to the external signal, and this can then modulate expression of *basS* and many other genes including the *arnB* operon that is implicated in LPS modification [18]. Overexpression of *basS* resulted in reduced susceptibility to colistin, and we also found that mutations in *basR* enabled cells to survive better under colistin stress (Figure 3). While mutations in BasSR are known to contribute to colistin susceptibility in *E. coli* [17,19], the high density of transposon mutants in our library allowed us to assess the impacts of mutations across this locus. We see that intra-genic insertions within *basS*, likely to alter expression of the rest of the gene, are beneficial, which has not been reported before. In *basS*, alternative potential start codons are present downstream of the small window of insertion sites we identified, suggesting that at least the first few amino acids of BasS are not required for colistin susceptibility [20]. Mutants with insertions which were oriented in the same direction as transcription of both *basR* and *eptA* also survived better than the wild type, further demonstrating the crucial importance of over-expression of *basS*. 

Transposon insertion within a small region of *hrpA* also enabled increased survival. HrpA is a DEAH-box ATP-dependent (RNA) helicase that has recently been shown to influence susceptibility to a range of antibiotics; mechanisms relating to RNA stability and ribosome interactions are proposed as possible contributors to this phenotype [21]. This work extends the potential impact of HrpA to include colistin and our data indicated that increased expression of the C-terminal domain of HrpA may be important (Figure 1B). Analysis of a *hrpA* deletion mutant gave inconclusive phenotypic results; this may not be unexpected, as TraDIS-Xpress data predicted only mutation of a defined region would be beneficial, rather than a complete knock-out mutant (as tested with the mutants drawn from the Keio collection). Moreover, TraDIS-Xpress is very sensitive and can identify mutants which have a small change in fitness upon competition, and not all mutants necessarily demonstrate a strong phenotype in isolated assays. It may be that HrpA has a modest impact on colistin susceptibility, or a specific mutation is needed for this to be evident. More work is needed to understand this, although our work suggests that this helicase may have an important role in colistin susceptibility. 

DNA repair protein genes (*uvrD* and *recF*) were also important in colistin susceptibility (Figure 1C) and in our validation experiments. Keio library knockouts showed decreased susceptibility to colistin compared with the wild type which was consistent with the TraDIS-Xpress data. Mutations in genes encoding DNA repair machinery appeared advantageous for survival under colistin stress. Whilst this may initially appear surprising, Kohanski et al. [16] have reported that a lack of DNA damage repair proteins (such as RecA) can lead to increased resistance to bactericidal antibiotics; we also observed a higher MIC of colistin for *recF* and *mutS* mutants (Figure 2). As helicases and DNA repair machinery were both involved in colistin susceptibility, we tested for synergy between colistin resistance and the topoisomerase inhibitors which impact nucleic acid conformation, but there was no phenotypic difference in response to colistin and ciprofloxacin between different helicase mutants from the Keio collection and the wild type *E. coli* BW25113 (Figure S5). 

Two other operons, *phn* and *fim*, were identified here as contributing to colistin susceptibility which have not been reported before. As colistin resistance can be caused by reducing the negative charge of LPS, the loss of function of the membrane components encoded by these operons might contribute to changing the charge of the outer membrane [17]. 

In our study, we also observed the role of small RNAs which are important in modulating the regulation of transcription and translation of many genes and have been implicated in multiple stress response conditions. Given the possible pleiotropic way in which small RNAs may function, it is not immediately clear which pathways and genes may be being modulated to impact colistin susceptibility, and further work is needed to explore this.

Although colistin was originally limited in its usage due to toxicity concerns, it has become one of the last lines of defense against multidrug-resistant bacteria and is a critically important antibiotic. Therefore, understanding the full spectrum of genes that contribute to susceptibility to colistin can help us understand how resistance can emerge and may be useful in designing future treatment regimens against multidrug-resistant pathogens. We were able to identify both genes known to contribute to colistin susceptibility, as well as a range of putative new loci at intragenic resolution. Through a single series of TraDIS-Xpress experiments (including a range of exposure conditions), we were able to produce all this information and identify concentration-dependent differences, demonstrating the power of this approach. In addition to identifying pathways that directly impact colistin susceptibility, TraDIS-Xpress can also identify pathways which, if inhibited, could impact colistin sensitivity. This can help the search for novel antibacterial combinations where colistin could be paired with an inhibitor that provides synergistic activity [22]. This approach promises to be very powerful in developing much needed new treatment regimens for Gram-negative multidrug-resistant pathogens.

In our data, up-regulation of *basS* had the most important role in colistin resistance, consistent with the literature [23]. Other loci were, however, also important, and we have also identified a putative role for *hrpA*, as well as a range of small RNAs and genes involved in DNA repair. The data presented here broaden the landscape of loci known to contribute to the survival of *E. coli* following exposure to colistin stress and will help to understand the development of colistin resistance in Gram-negative pathogens where this drug remains of great importance. This study focused on an *E. coli* K-12 strain which is a model organism and not necessarily representative of isolates in circulation. However, colistin susceptibility mechanisms known to date are generally part of the core genome and the mechanisms identified in this study are likely to be applicable to other *E. coli* and Gram-negative pathogens but cannot capture all possible important genes in other strains and species [24].

## 4. Materials and Methods

### 4.1. TraDIS-Xpress Library

The large transposon mutant library of *E. coli* strain BW25113 described in Yasir et al. [15] was used in this study. Briefly, the transposon (a mini-Tn5 coding for kanamycin resistance (*aph(3’)-Ia*)) incorporates an outward-transcribing *tac* promoter 3’ to the kanamycin resistance gene. The promoter is inducible by isopropyl-β-D-thiogalactopyranoside (IPTG), allowing over-expression or repression of genes (depending on the transposon insertion site and orientation), as well as gene inactivation. This approach allows the roles of both essential and non-essential genes to be assessed following exposure to stress.

### 4.2. Colistin Exposure Conditions and TraDIS-Xpress Sequencing

The MIC of colistin for BW25113 was determined in Lysogeny broth (LB) (Fisher Bioreagents BP9722-500) using the broth dilution method and LB was used for the TraDIS-Xpress experiment. For use in TraDIS-Xpress, approximately 10^7^ cells from the mutant library were inoculated into LB at a range of doubling concentrations of colistin sulfate (Sigma-Aldrich, St. Louis, MO, USA), starting from 0.125 μg/mL to 1 μg/mL (0× MIC [control], 0.25× MIC, 0.5× MIC, 1× MIC, 2× MIC). We used IPTG (Sigma-Aldrich, Rehovot, Israel) stock of one molar concentration (1 M) to induce the cultures of transposon mutant library at 0.2 mM and 1 mM as required (Table S1). Experiments were performed with no induction, or with induction using 0.2 mM or 1 mM IPTG. Cultures were incubated overnight at 37 °C. All experiments were performed in duplicate to give a total of 30 independent TraDIS-Xpress experiments.

After growth in experimental conditions, DNA was extracted from pools of mutants using a ‘Quick-DNA™’ Fungal/Bacterial 96 kit extraction kit (Zymo Research, Irvine, CA, USA). DNA was then fragmented using a Nextera DNA library preparation kit (Illumina, San Diego, CA, USA) with the modification that Tnp-i5 oligonucleotides were used instead of i5 index primers, with 28 PCR cycles [15]. The resulting DNA was size selected to purify fragments between 300 and 500 bp and sequenced on an Illumina NextSeq 500 sequencing instrument using a NextSeq 500/550 High Output v2 kit (75 cycles).

### 4.3. Bioinformatics

Results were analyzed using Bio-TraDIS (version 1.4.1) [25] and our in-house AlbaTraDIS software (version 1.0.4) [26] developed specifically for TraDIS-Xpress analysis. Briefly, Bio-TraDIS was used to align sequence reads against the BW25113 reference genome (CP009273) using SMALT (version 0.7.4). From these results, tab delimited insertion site files were produced listing the number of insertions per gene on the forward and reverse strand, to allow for visualization and manual interpretation in the genome browser Artemis.

The patterns of inserts were compared between colistin exposed and control conditions. AlbaTraDIS then calculated the number of inserts within each gene, as well as assessing the number of ‘forward’ and ‘reverse’ insertions per gene and within the 198 bp window upstream and downstream of each gene. The number of sequence reads was modelled on a per-gene basis using a negative binomial distribution and an adapted exact test as implemented in edgeR [27], followed by a correction for multiple testing [28] to identify significant differences in reads mapping to each gene between control and colistin stress conditions. A set of default cut-offs for significance and number of reads were applied (*q*-value ≤ 0.05, logFC ≥ 1, logCPM > 8). The resulting gene list provided a prediction of the genes involved in colistin survival, where the only difference between stress and control condition is the presence of colistin which limits any selection for mutants adapted to the media and growth conditions. AlbaTraDIS also predicted that changes in expression of a gene (either down- or up-regulation) influence survival. The insertion patterns at candidate loci were visually inspected using ‘Artemis’ which was also used to capture images for figures [29].

### 4.4. Validation Experiments

A total of 18 mutants in duplicate were selected from the Keio collection of mutants to validate predictions about sensitivity to colistin made using TraDIS-Xpress [30]. These included genes for the two-component system, DNA repair mechanisms and transporter membranes identified as major contributors to colistin sensitivity. The *E. coli* Keio collection (Dharmacon Horizon Discovery) is supplied with duplicate pairs of each mutant, and both were tested for their sensitivity to colistin by MIC determination. The MIC was determined using the plate MIC method by Agarwal et al. [31]. All experiments were duplicated, giving at least four measurements for each gene (two repeats from each of the two mutant alleles of each gene). Wild-type BW25113 was included in all experiments as a control. 

We used growth curves to find out the added advantage of *basS* gene overexpression in the presence of colistin. *E. coli* BW25113 containing either pJMA/*basS* or pJMA empty vector was grown under colistin stress in LB broth with different induction concentrations (0.01%, 0.02% and 0.1%) of rhamnose (Sigma-Aldrich, Steinheim, Germany) and incubated at 37 °C overnight in FluoStar Omega (BMG LabTech, Guelph, ON, Canada) for optical density (OD) measurements at 600 nm wavelength.

### 4.5. Comparing Combinations of Two Antibiotics at Different Concentrations

We used a checkerboard experimental design to investigate synergy between colistin and ciprofloxacin in a 96-well microtiter plate. Each well contained a final volume of 100 μL. The highest concentration for ciprofloxacin was 0.25 μg/mL in column 2, followed by a series of 50% dilutions in each well from column 3 to column 12. The highest concentration for colistin was 2 μg/mL in row A, followed by a series of 50% dilutions in each well from row B to G (Table S4). Column 1 did not contain ciprofloxacin, and row H did not contain colistin. Well H1 had neither of the antibiotics. The plate was sealed with a permeable cover after inoculation with ~10^4^ cells of *E. coli* BW25113 to each well and incubated at 37 °C overnight. 

## Figures and Tables

**Table 1:** Pathways and genes with significant differences in transposon insertions after colistin exposure. Values indicate logFC.

Pathway	Gene	Annotation	Multiple of MIC			
			2×	1×	0.5×	0.25×
Lipid biosynthesis and regulation	*basR*	response regulator in two-component regulatory system with BasS	4.03	0.00	0.00	0.00
	*basS*	sensory histidine kinase in two-component regulatory system with BasR	17.44	5.65	1.94	5.45
	*waaB*	UDP-D-galactose:(glucosyl)lipopolysaccharide-1 and 6-D-galactosyltransferase	0.00	−1.82	0.00	0.00
	*waaC*	ADP-heptose:LPS heptosyl transferase I	0.00	−2.25	−1.70	0.00
	*waaG*	glucosyltransferase I	0.00	−3.06	−1.98	−1.61
	*waaP*	kinase that phosphorylates core heptose of lipopolysaccharide	0.00	−2.46	−1.92	0.00
	*waaR*	UDP-D-galactose:(glucosyl)lipopolysaccharide-alpha-1 and 3-D-galactosyltransferase	0.00	−3.83	−2.01	−1.11
	*waaS*	lipopolysaccharide core biosynthesis protein	0.00	−1.96	−1.63	−1.60
	*waaU*	lipopolysaccharide core biosynthesis	0.00	−1.10	0.00	0.00
	*lptC*	lipopolysaccharide export and IM-tethered periplasmic protein of the LptBFGC export complex	0.00	1.57	1.50	1.42
	*waaD*	ADP-L-glycero-D-mannoheptose-6-epimerase and NAD(P)-binding	0.00	−2.66	−2.16	0.00
	*wbbH*	O-antigen polymerase	0.00	−1.14	−1.16	−1.17
	*wbbI*	d-Galf:alpha-d-Glc beta-1 and 6-galactofuranosyltransferase	0.00	0.00	−1.08	0.00
	*wbbK*	lipopolysaccharide biosynthesis protein	0.00	−1.59	−1.45	−1.57
Transcription and translation regulation	*aspU*	tRNA-Asp	0.00	0.00	0.00	−2.70
	*cmoB*	tRNA (cmo5U34)-carboxymethyltransferase and carboxy-SAM-dependent	0.00	1.30	0.00	0.00
	*valS*	valyl-tRNA synthetase	0.00	0.00	0.00	−3.15
	*truA*	tRNA pseudouridine(38–40) synthase	0.00	1.11	1.32	1.23
	*hfq*	global sRNA chaperone; HF-I and host factor for RNA phage Q beta replication	0.00	1.41	1.55	1.55
	*ohsC*	sRNA antisense regulator of shoB toxin	0.00	−2.49	−2.81	−2.55
	*oxyS*	sRNA antisense regulator activates genes that detoxify oxidative damage and Hfq-dependent	0.00	0.00	−2.27	0.00
	*rpoN*	RNA polymerase and sigma 54 (sigma N) factor	0.00	1.99	0.00	1.81
	*rydC*	sRNA antisense regulator of yejABEF and Hfq-dependent; over-expression causes a thermosensitive growth phenotype on minimal glycerol and maltose and or ribose media	0.00	−2.31	0.00	−1.83
	*appY*	global transcriptional activator; DLP12 prophage	0.00	−1.66	−1.66	−1.69
Membrane associated genes	*bhsA*	biofilm and cell surface and signaling protein	0.00	−1.86	0.00	−2.21
	*ariR*	RcsB connector protein for regulation of biofilm and acid-resistance	0.00	−1.04	−1.26	−1.23
	*ompA*	outer membrane protein A (3a;II*;G;d)	0.00	−1.32	0.00	0.00
	*surA*	peptidyl-prolyl cis-trans isomerase (PPIase)	0.00	−3.29	0.00	−1.38
	*flgA*	assembly protein for flagellar basal-body periplasmic P ring	0.00	1.22	0.00	0.00
Transporters	*glpT*	sn-glycerol-3-phosphate transporter	3.26	1.08	1.15	1.14
	*phnE*	4312259_4313047	3.84	1.44	1.35	1.29
	*phnF*	putative DNA-binding transcriptional regulator of phosphonate uptake and biodegradation	4.14	1.05	1.02	0.00
	*ygcS*	putative transporter	12.28	0.00	0.00	0.00
DNA associated proteins	*hrpA*	putative ATP-dependent helicase	16.12	2.80	0.00	2.99
	*recF*	gap repair protein	7.68	4.60	0.00	0.00
	*uvrD*	DNA-dependent ATPase I and helicase II	8.10	0.00	0.00	0.00
	*dinI*	DNA damage-inducible protein I	0.00	0.00	0.00	−1.28
	*recG*	ATP-dependent DNA helicase	0.00	10.51	4.98	0.00
	*dcd*	2′-deoxycytidine 5′-triphosphate deaminase	0.00	3.54	2.94	3.74
	*dnaG*	DNA primase	0.00	−4.65	−3.74	0.00
	*uspC*	universal stress protein	0.00	−1.14	−1.09	0.00
	*seqA*	negative modulator of initiation of replication	0.00	1.73	1.74	0.00
Miscellaneous	*ybbC*	putative immunity protein	0.00	−2.21	−2.03	−2.08
	*ybcV*	DLP12 prophage; uncharacterized protein	0.00	−1.74	−1.37	−1.48
	*ydcD*	uncharacterized protein	0.00	−2.86	0.00	0.00
	*ygeN*	2988672_2989379	0.00	−1.84	−1.87	−1.76
	*yjbL*	uncharacterized protein	0.00	−2.44	−2.44	−2.48
	*yjbS*	uncharacterized protein	0.00	−2.22	−2.04	−2.39
	*yfcZ*	UPF0381 family protein	0.00	2.99	0.00	0.00
	*ycjS*	putative NADH-binding oxidoreductase	0.00	1.19	1.09	1.19

## Data Availability

All sequence data supporting this study have been deposited under the accession number E-MTAB-11807 and the control data are deposited under the accession number E-MTAB-11808.

## Supplementary Materials

The following supporting information can be downloaded at: https://www.mdpi.com/article/10.3390/antibiotics11111668/s1, Figure S1: Scatter chart showing the number of sequence-reads per gene; Figure S2: Example of differential expression controlled by IPTG concentration; Figure S3: Growth curves of *E. coli* BW25113 containing pJMA/basS or pJMA empty vector under colistin stress; Figure S4: Assessment of colistin susceptibility of defined mutants in isolation; Figure S5. Summary of checkerboards of colistin and ciprofloxacin of *E. coli* BW25113 and Keio mutants; Table S1: Experimental treatments; Table S2: Sequencing run statistics; Table S3: Correlation values of replicate 1 and replicate 2 of control and the colistin stress conditions; Table S4: Colistin and ciprofloxacin checkerboard setup.
