# Supplementary material for: Genome-Wide Analysis of Innate Susceptibility Mechanisms of Escherichia coli to Colistin
Source: Antibiotics (Basel). 2022 Nov 21;11(11):1668. doi: 10.3390/antibiotics11111668 (PMC9687012; doi:10.3390/antibiotics11111668)
Supplement: Supplementary file 1 [file antibiotics-11-01668-s001.zip › antibiotics-1954289-supplementary.pdf]

SUPPLEMENTARY MATERIAL

Genome-Wide Analysis of Innate Susceptibility Mechanisms of *Escherichia coli* to Colistin

Muhammad Yasir [1,*,†], A. Keith Turner [1,†], Sarah Bastkowski [1], Martin Lott [1], Emma R. Holden [1], Andrea Telatin [1], Andrew J. Page [1], Mark A. Webber [1,2] and Ian G. Charles [1,2]

[1] Quadram Institute Bioscience, Rosalind Franklin Road, Norwich NR4 7UQ, UK
[2] Norwich Medical School, Norwich Research Park, Colney Lane, Norwich NR4 7TJ, UK
* Correspondence: muhammad.yasir@quadram.ac.uk; Tel.: +44-1603255391
† These authors contributed equally to this work.

Table S1: Experimental treatments

Concentration	0 mM IPTG	0.2 mM IPTG	1 mM IPTG
2 x MIC colistin	2 x MIC colistin with 0 mM IPTG	2 x MIC colistin with 0.2 mM IPTG	2 x MIC colistin with 1 mM IPTG
1 x MIC colistin	1 x MIC colistin with 0 mM IPTG	1 x MIC colistin with 0.2 mM IPTG	1 x MIC colistin with 1 mM IPTG
0.5 x MIC colistin	0.5 x MIC colistin with 0 mM IPTG	0.5 x MIC colistin with 0.2 mM IPTG	0.5 x MIC colistin with 1 mM IPTG
0.25 x MIC colistin	0.25 x MIC colistin with 0 mM IPTG	0.25 x MIC colistin with 0.2 mM IPTG	0.25 x MIC colistin with 1 mM IPTG
0 x MIC colistin (control)	0 x MIC colistin with 0 mM IPTG	0 x MIC colistin with 0.2 mM IPTG	0 x MIC colistin with 1 mM IPTG

MIC = minimal inhibitory concentration. IPTG = isopropyl β- d-1-thiogalactopyranoside.

There were two replicates of each treatment combination

Table S2: Sequencing run statistics

Sample	Total reads	Reads matched	% Matched	Reads mapped	% Mapped	Total unique insert sites	Average distance (bp) between inserts
controlLBrep3-1mM_S44_L999_R1_001.fastq	6031559	3033767	50	1910628	63	347558	13
controlLBrep3-0.2mM_S57_L999_R1_001.fastq	8738674	3655412	42	1898899	52	184777	25
controlLBrep3_S49_L999_R1_001.fastq	8632256	3626215	42	1783687	49	195998	24
controlLBrep4-1mM_S37_L999_R1_001.fastq	9564275	4296700	45	2413707	56	298137	16
controlLBrep4-0.2mM_S39_L999_R1_001.fastq	8180854	3552001	43	1923180	54	230307	20
controlLBrep4_S40_L999_R1_001.fastq	9076061	3904669	43	1906172	49	197515	23
Colistinrep1-25MIC1mM_S31_L999_R1_001.fastq	9211081	6208889	67	4497436	72	658559	7
Colistinrep1-25MIC-0.2mM_S34_L999_R1_001.fastq	9502606	6393804	67	4635996	73	674266	7
Colistinrep1-25MIC_S35_L999_R1_001.fastq	9163212	6360876	69	4295149	68	669871	7

Colistinrep1-2MIC1mM_S44_L999_R1_001.fastq	6216521	4029798	65	2883518	72	60061	77
Colistinrep1-2MIC-0.2mM_S45_L999_R1_001.fastq	6146899	4131620	67	3108991	75	35224	131
Colistinrep1-2MIC_S38_L999_R1_001.fastq	7069947	4986969	71	3820292	77	34077	136
Colistinrep1-5MIC1mM_S51_L999_R1_001.fastq	10241441	7253316	71	5406212	75	677024	7
Colistinrep1-5MIC-0.2mM_S40_L999_R1_001.fastq	10005681	7056284	71	5299664	75	701711	7
Colistinrep1-5MIC_S47_L999_R1_001.fastq	8692272	6257023	72	4353615	70	664703	7
Colistinrep1-MIC1mM_S52_L999_R1_001.fastq	7646978	5425713	71	4157783	77	659600	7
Colistinrep1-MIC-0.2mM_S49_L999_R1_001.fastq	8596805	5962307	69	4531253	76	681525	7
Colistinrep1-MIC_S50_L999_R1_001.fastq	8180011	5879998	72	4188011	71	628443	7

Colistinrep2-25MIC1mM_S39_L999_R1_001.fastq	9967776	6922029	69	5402897	78	669271	7
Colistinrep2-25MIC-0.2mM_S40_L999_R1_001.fastq	10191470	7022432	69	5493519	78	736784	6
Colistinrep2-25MIC_S37_L999_R1_001.fastq	9996200	7048236	71	5076933	72	697001	7
Colistinrep2-2MIC1mM_S42_L999_R1_001.fastq	7461190	5228786	70	4021881	77	117474	39
Colistinrep2-2MIC-0.2mM_S49_L999_R1_001.fastq	6331193	4404349	70	3378050	77	52842	88
Colistinrep2-2MIC_S50_L999_R1_001.fastq	7568278	5420026	72	4547368	84	42988	108
Colistinrep2-5MIC1mM_S31_L999_R1_001.fastq	10658169	7327714	69	5685610	78	707201	7
Colistinrep2-5MIC-0.2mM_S30_L999_R1_001.fastq	13819391	9128953	66	6980634	76	735041	6
Colistinrep2-5MIC_S32_L999_R1_001.fastq	10319868	7079792	69	5064250	72	720294	6

Colistinrep2-MIC1mM_S45_L999_R1_001.fastq	9273321	6605435	71	5186338	79	686625	7
Colistinrep2-MIC-0.2mM_S51_L999_R1_001.fastq	9922337	7115673	72	5597110	79	709629	7
Colistinrep2-MIC_S43_L999_R1_001.fastq	7704930	5555566	72	3989807	72	674875	7

Table S3: Correlation values of replicate 1 and replicate 2 of control and the colistin stress conditions

Sample	Correlation value (r)
Control	0.994883728
Colistin 2MIC	0.98784257
Colistin 1MIC	0.99995168
Colistin 0.5MIC	0.999998307
Colistin 0.25MIC	0.999805737

Table S4: Colistin and ciprofloxacin checkerboard setup

	1	2	3	4	5	6	7	8	9	10	11	12
A	4	4	4	4	4	4	4	4	4	4	4	4
		0.25	0.125	0.06	0.03	0.015	0.008	0.004	0.002	0.001	0.0005	0.00025
B	2	2	2	2	2	2	2	2	2	2	2	2
		0.25	0.125	0.06	0.03	0.015	0.008	0.004	0.002	0.001	0.0005	0.00025
C	1	1	1	1	1	1	1	1	1	1	1	1
		0.25	0.125	0.06	0.03	0.015	0.008	0.004	0.002	0.001	0.0005	0.00025
D	0.5	0.5	0.5	0.5	0.5	0.5	0.5	0.5	0.5	0.5	0.5	0.5
		0.25	0.125	0.06	0.03	0.015	0.008	0.004	0.002	0.001	0.0005	0.00025
E	0.25	0.25	0.25	0.25	0.25	0.25	0.25	0.25	0.25	0.25	0.25	0.25
		0.25	0.125	0.06	0.03	0.015	0.008	0.004	0.002	0.001	0.0005	0.00025
F	0.125	0.125	0.125	0.125	0.125	0.125	0.125	0.125	0.125	0.125	0.125	0.125
		0.25	0.125	0.06	0.03	0.015	0.008	0.004	0.002	0.001	0.0005	0.00025
G	0.062	0.062	0.062	0.062	0.062	0.062	0.062	0.062	0.062	0.062	0.062	0.062
		0.25	0.125	0.06	0.03	0.015	0.008	0.004	0.002	0.001	0.0005	0.00025
H												
		0.25	0.125	0.06	0.03	0.015	0.008	0.004	0.002	0.001	0.0005	0.00025

Colistin concentrations are highlighted in blue and ciprofloxacin concentrations are highlighted in yellow.

Supplementary Figures

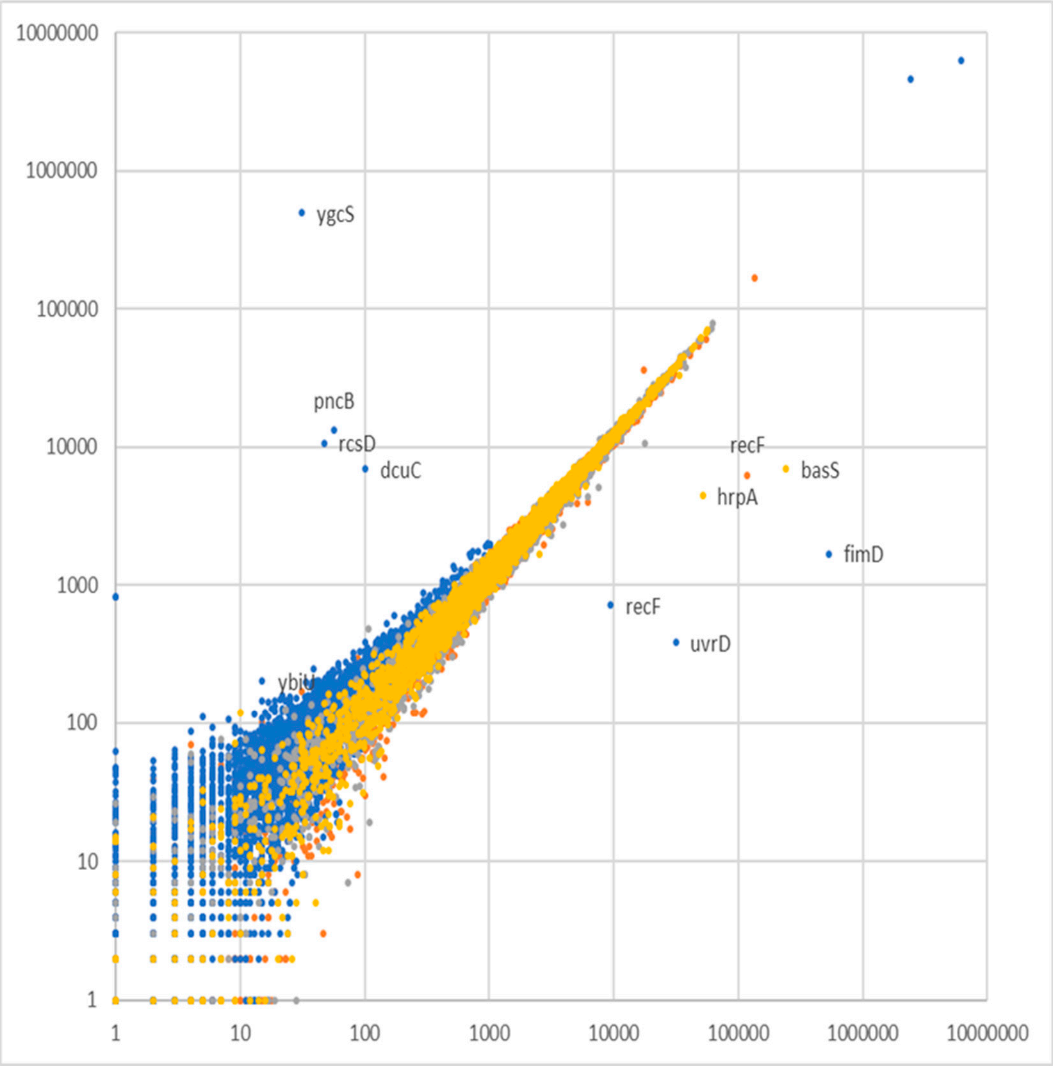

Figure S1. Scatter chart showing the number of sequence-reads per gene.

Each point on the chart represents a gene. For each gene, the number of sequence-reads obtained in control replicate 1 is plotted against the number in control replicate 2 and shown in black; the number of sequence-reads obtained in colistin replicate 1 are plotted against the number in colistin replicate 2 under growth conditions of 0.25 x MIC, 0.5 x MIC, 1 x MIC and 2 x MIC which are shown in grey, orange, yellow and blue, respectively. This shows the degree of experimental variation between biological replicates. MIC = minimum inhibitory concentration.

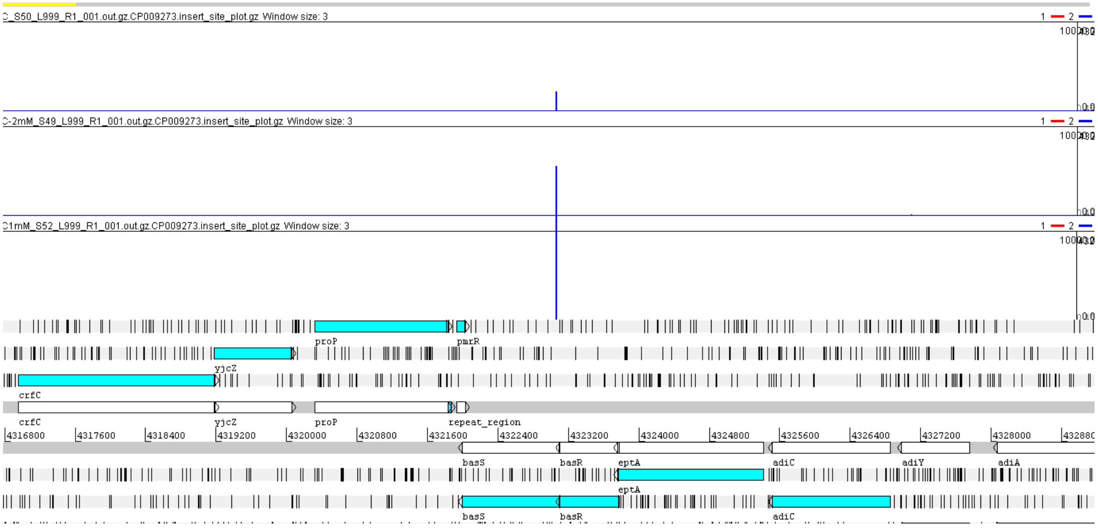

Figure S2. Example of differential expression controlled by IPTG concentration

Differential selection of insert sites within *basS* depending on promoter induction concentration. Above the genome representation, vertical blue lines indicate the positions of mapped reads; the height of the bar represents the relative number of reads mapped. The scale is set to minimum of 10,000 reads to highlight IPTG dependent induction. The top row shows uninduced culture, middle row shows 0.2mM induction of IPTG and the bottom row shows 1mM induction. All samples shown were exposed to 1X the MIC concentration of colistin.

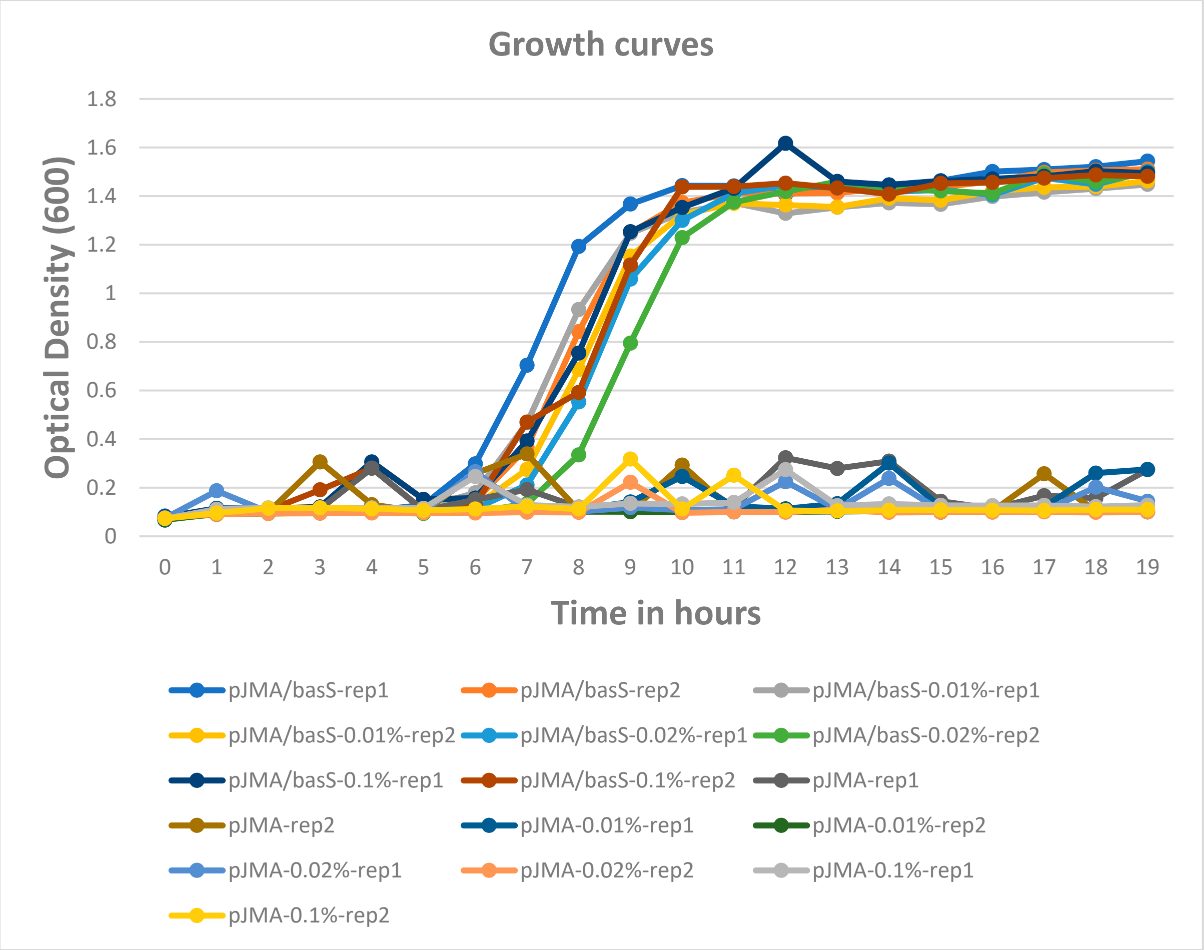

Figure S3. Growth curves of *E. coli* BW25113 containing pJMA/basS or pJMA empty vector under colistin stress

E. coli BW25113 containing either pJMA/basS or pJMA empty vector was grown in colistin stress with different induction concentrations 0.01%, 0.02% and 0.1% of rhamnose and incubated at 37°C for overnight. *E. coli* BW25113 containing pJMA/basS has shown growth curves at all induction levels including no induction while *E. coli* BW25113 containing pJMA empty vector have not been able to grow.

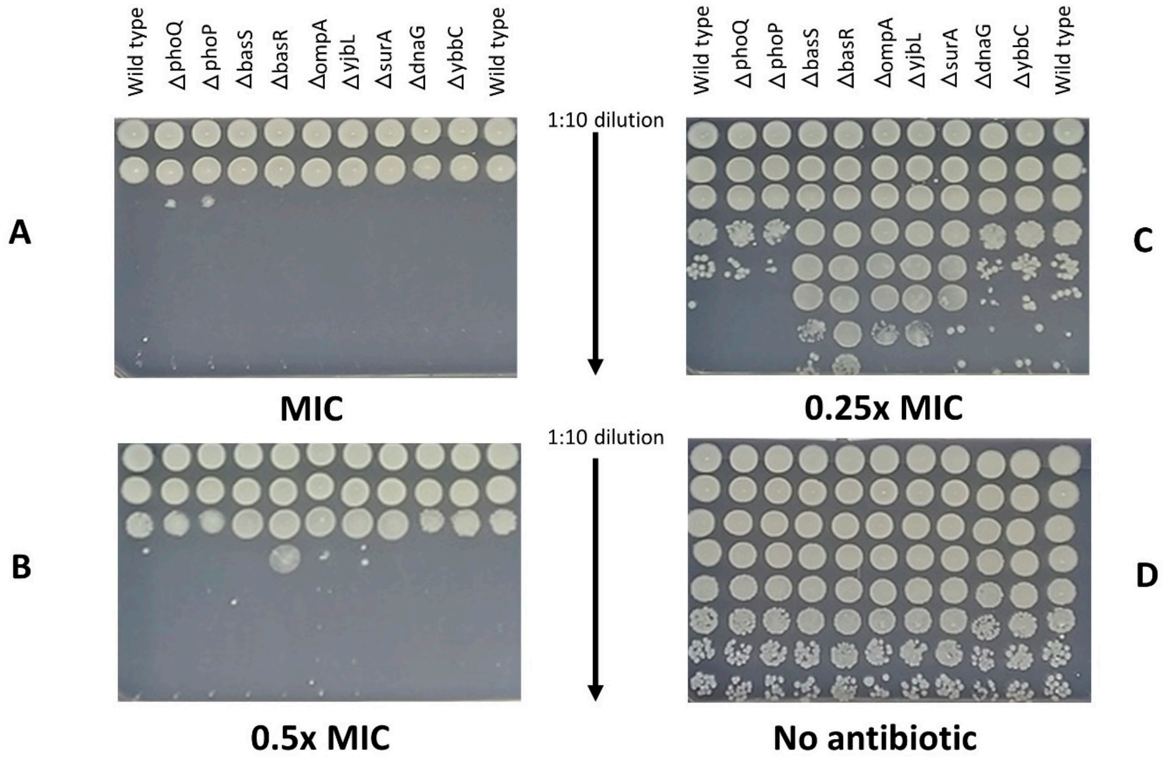

Figure S4. **Assessment of colistin susceptibility of defined mutants in isolation**
Mutants and the wild type overnight cultures were serially diluted (from top row down) onto an LB agar plates containing (A) the minimal inhibitory concentration (MIC), (B) half of the MIC (0.5x MIC) and (C) a quarter of the MIC (0.25x MIC) of colistin and (D) no colistin and incubated overnight at 37 °C.

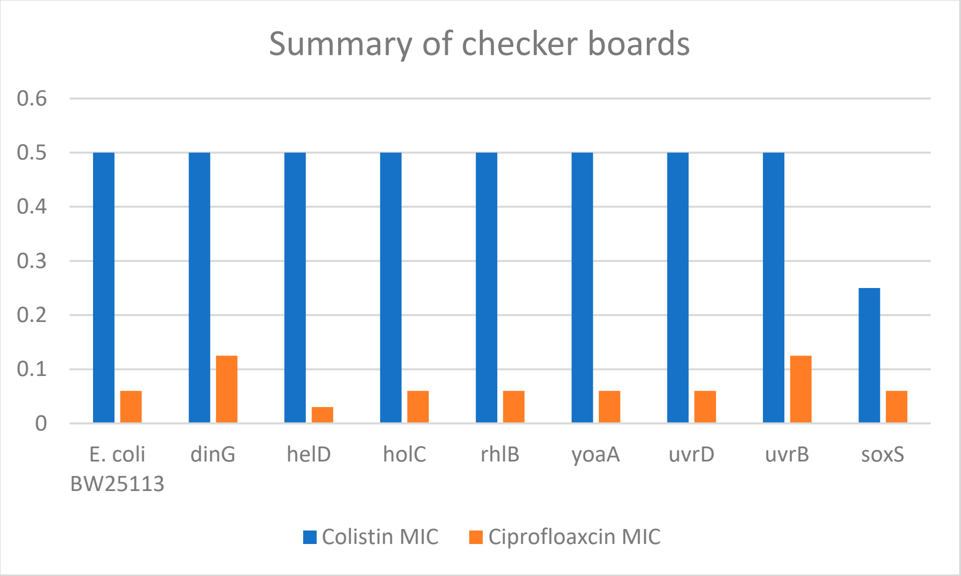

Figure S5. Summary of checkerboards of colistin and ciprofloxacin of *E. coli* BW25113 and Keio mutants

The checkerboard was setup using concentration of colistin and ciprofloxacin as explained in Table S4. *E. coli* BW25113 and different Keio mutants (*dinG, held, holC, rhlB, uvrD, uvrB and soxS*) were inoculated in individual checkerboard plates and results were interpreted by growth or no growth using visual observation. MICs of colistin and ciprofloxacin is plotted as blue and orange bars respectively.
